# Less reduction of psychosocial problems among adolescents with unmet communication needs

**DOI:** 10.1007/s00787-016-0901-y

**Published:** 2016-09-13

**Authors:** Margot Jager, Sijmen A. Reijneveld, Josue Almansa, Janneke Metselaar, Erik J. Knorth, Andrea F. De Winter

**Affiliations:** 1 Department of Health Sciences, University Medical Center Groningen, University of Groningen, Antonius Deusinglaan 1/FA10, 9713 AV Groningen, The Netherlands; 2 Department of Special Needs Education and Youth Care, University of Groningen, Groningen, The Netherlands

**Keywords:** Adolescent, Psychosocial problems, Patient-centered communication, Shared decision-making, Treatment adherence, Mediation

## Abstract

Patient-professional communication has been suggested to be a major determinant of treatment outcomes in psychosocial care for children and adolescents. However, the mechanisms involved are largely unknown and no longitudinal studies have been performed. Our aim was, therefore, to assess over the course of 1 year, the impact of patient-centered communication on psychosocial problems of adolescents in psychosocial care, including the routes mediating this impact. We obtained data on 315 adolescents, aged 12–18 years, enrolled in child and adolescent social or mental health care. We assessed patient-centered communication by comparing the needs and experiences of adolescents with regard to three aspects of communication: affective quality, information provision, and shared decision-making. Changes in psychosocial problems comprised those reported by adolescents and their parents between baseline and 1 year thereafter. Potential mediators were treatment adherence, improvement of understanding, and improvement in self-confidence. We found a relationship between unmet needs for affective quality, information provision, and shared decision-making and less reduction of psychosocial problems. The association between the unmet need to share in decision-making and less reduction of psychosocial problems were partially mediated by less improvement in self-confidence (30 %). We found no mediators regarding affective quality and information provision. Our findings confirm that patient-centered communication is a major determinant of treatment outcomes in psychosocial care for adolescents. Professionals should be aware that tailoring their communication to individual patients’ needs is vital to the effectiveness of psychosocial care.

**Electronic supplementary material:**

The online version of this article (doi:10.1007/s00787-016-0901-y) contains supplementary material, which is available to authorized users.

## Introduction

An estimated 10–25 % of adolescents have one or more psychosocial problems: emotional, behavioral, and/or social problems [1–5]. These problems may have a major impact on the daily life of adolescents and their families, indicating a need for early and effective treatment. As patients who receive psychosocial care sometimes do not participate actively and do not always finish treatment, psychosocial problems fairly often remain [6, 7]. Patient-professional communication is at the core of psychosocial care and has been suggested to be a major determinant of treatment outcomes in such care for children and adolescents [8, 9]. However, mechanisms leading to this are largely unknown and no longitudinal studies have been performed.

Patient-centered communication has been shown to be associated with improvements in adherence, satisfaction, and health outcomes in various types of health care such as general practice, oncology and diabetes care [10–15]. Patient-centered communication includes the active involvement of patients in the care process and regard for their individual needs and preferences [16, 17]. In a previous study we found that adolescents’ experiences in psychosocial care did not always match their needs with regard to three pivotal domains of communication: affective quality of the communication, information provision, and shared decision-making [18]. Furthermore, unmet communication needs were, over a period of 3 months, associated with poorer treatment adherence, less improvement of understanding, and less improvement in self-confidence, although patterns varied across the afore-mentioned domains [18].

To gain more insight into the role of patient-centered communication in the psychosocial care process, we aim to assess, over the course of 1 year, the impact of patient-centered communication on psychosocial problems of adolescents in psychosocial care, and the routes mediating this impact.

To answer our research questions we followed adolescents and their parents during 1 year, starting from the moment they entered the psychosocial care system. We performed separate analyses for each of the three important communication functions: affective quality, information provision, and shared decision-making. Our study provides for professionals in adolescent psychosocial care a better understanding of how patient-centered communication plays in the care process, including the importance of tailoring communication to individual patients’ needs to improve outcomes of care.

## Method

### Study design

We conducted this study within the framework of TakeCare, a large longitudinal prospective cohort study designed to investigate the trajectories in and outcomes of care for children and adolescents with emotional and behavioral problems [19]. The study includes all new cases entering psychosocial care organizations in one Dutch region. The sources of our data were adolescents, parents, and professionals. This report is based on data from the first (T1, before psychosocial care started), the second (T2, 3 months after T1), and the third (T3, 1 year after T1) measurement waves, which ran from April 2011 through June 2014.

We obtained informed consent from all participating adolescents (and their parents if the adolescent was below the age of 16). The study was approved by the Medical Ethical Board of the University Medical Center Groningen.

### Sample and procedure

Adolescents (12–18 years old) who signed up for psychosocial care in three organizations for child and adolescent social care or child and adolescent mental health care received written information about the study (*n* = 766). Of the potentially eligible participants, 141 refused to receive further information about the study by completing the opt-out form that was attached to the written introduction. Of the 625 remaining eligible participants, 416 (67 %) were reached and willing to participate in the study [19]. Participants then received a questionnaire, by e-mail or on paper, depending on the preference of the participant. If needed, telephone interviews or home visits were arranged.

This study included adolescents who filled in questions as to how relevant they considered communication to be at the first (T1) measurement wave, and their actual experiences with communication at the second (T2) or third (T3) measurement wave (*n* = 315; 76 % of baseline sample). Reasons given for not reporting communication experiences were: treatment was aimed at parents or other family members, treatment did not start after all, or questionnaires were not returned. Our study sample (*n* = 315) did not differ from the total sample (*n* = 416) regarding adolescent and family characteristics, care-related characteristics, and psychosocial problems.

### Measures

#### Independent variables

The independent variables in this study were three aspects of *patient*-*centered communication:* affective quality, information provision, and shared decision-making, measured using an adapted version of the Consumer Quality Index (CQI) [20]. The CQI assesses both the attributed relevance of and experiences with different aspects of care. Items concerning patient-professional communication were derived from three existing CQI versions that have been used in preventive child health care [21], outpatient mental health care [22, 23], and outpatient occupational therapy [24].

*Relevance attributed to communication* was assessed by asking adolescents to rate how important they considered communication across three domains (1 ‘not important’ to 4 ‘very important’). Nine items assessed affective quality of the communication (Cronbach’s α for study sample (*n* = 315): 0.89), five items assessed information provision (Cronbach’s α: 0.84), and six items assessed shared decision-making (Cronbach’s α: 0.71). The full list of items can be found in Supplement 1.

*Experiences with communication* were assessed after 3 months (T2) for adolescents who had started their care trajectory almost immediately after T1, and after 1 year (T3) for adolescents who started their care trajectory after T2. Adolescents rated the same items again, now assessing their actual experiences (1 ‘No’ to 4 ‘Yes’). If respondents rated an item on the experience scale with code 5 (no experience/don’t know) this item was counted as a missing value [22].

*Attributed relevance—actual experience discrepancies* were determined based on the afore-mentioned questionnaires. First, relevance scores were dichotomized as either important (highest 75 %) or less important (lowest 25 %). Second, experience scores were dichotomized in the same way, as either experienced (highest 75 %) or less experienced (lowest 25 %). Third, relevance scores and experience scores were combined into three categories in which the two types of discrepancy were separated: (1) agreement (less important—less experienced, or important—experienced), (2) important—less often experienced, and (3) less important—experienced. These three steps were performed separately for affective quality, information provision and shared decision-making, resulting in three categorical variables that express how well patients’ experiences matched the relevance they attributed to the communication aspects, i.e., the level of patient-centeredness.

#### Dependent variables

The main outcome variable concerned 1-year changes in adolescents’ psychosocial problems. These were assessed using the Dutch self-report and parent-report versions of the Strengths and Difficulties Questionnaire (SDQ) [25, 26]. The SDQ contains 25 items describing positive and negative attributes of adolescents. The items are scored as follows: 0 = not true; 1 = somewhat true; 2 = certainly true, on the basis of the preceding 6 months at baseline and of the preceding month at follow-up. The SDQ consists of five scales of five items each: emotional symptoms, conduct problems, hyperactivity/inattention, peer problems, and prosocial behavior. Scores for the first four scales add up to a total difficulties score (TDS) ranging from 0 to 40, with higher scores indicating more problems. TDSs of adolescents and their parents were added and divided by two, resulting in a mean TDS at baseline (T1) and a mean TDS after 1 year (T3).

Changes in adolescents’ psychosocial problems were assessed by adjusting adolescents’ TDS after 1 year as compared to their TDS at the start of the care trajectory. Higher scores on the dependent variable indicated less reduction of psychosocial problems after 1 year.

#### Mediating variables

Potential mediating variables were treatment adherence, improvement of understanding, and improvement in self-confidence. *Treatment adherence* was assessed by asking the most involved professional to what extent they agreed with the statement: “The adolescent demonstrated adherence”. We illustrated this statement with examples: fulfilling agreements, following recommendations, carrying out homework assignments, or taking prescribed medication.

*Improvement of understanding* was assessed by asking the professional, as well as the adolescent, how much they thought the adolescent had learned so far due to psychosocial care. We illustrated this question with examples: better understanding of the problems, and knowing how to handle difficult situations.

*Improvement in self*-*confidence* was assessed by asking the professional and the adolescent whether the feelings of the adolescent had changed positively because of psychosocial care. This question was also illustrated with examples: improved self-confidence, worrying less, and feeling less hopeless.

Answers on all three questions were given on a Likert scale from 0 (absolutely not) to 10 (very much). Professional and patient ratings of improvement of understanding were combined by adding up the scores and dividing this new score by two, resulting in a mean score. The same was done to calculate mean scores regarding improvement in self-confidence.

Similar to adolescents’ communication experiences, mediating variables were assessed after 3 months (T2) or after 1 year (T3), depending on the start of the care trajectory.

#### Participant characteristics

Participant characteristics included adolescent and family characteristics and care-related characteristics. *Adolescent and family characteristics* involved age, gender, ethnicity, family composition, and parental employment. Ethnicity was defined as non-Dutch if the adolescent or at least one of his/her biological parents was born outside the Netherlands. Family composition was dichotomized as two-parent family (both biological parents live with the adolescent) vs. other (e.g. one-parent family, separated parents, foster care, residential home). Parental employment was defined as employed if at least one of the parents had a paid job.

*Care*-*related characteristics* included the care setting and the care and treatment trajectory. The care setting referred to either social or mental health care for children and adolescents. In child and adolescent social care most professionals were social workers or family workers. In child and adolescent mental health care, the professionals were usually psychologists, psychiatrists or psychotherapists. Duration of care and treatment was defined as either less or more than 6 months.

### Statistical analyses

#### Multiple imputation of missing data

To achieve good efficiency of estimation and sufficient statistical power, variables with the largest amount of missing values were imputed through multiple imputation techniques [27, 28]. Data were missing for some self-reported and parent-reported total difficulties scores of the SDQ (8.6 and 20.0 % for T1 and T3, respectively), professional-reported treatment adherence (14.3 %), and parental employment (8.6 %). These missing values were imputed ten times based on the regression method, using as predictors the other variables included in the model. The imputed datasets were then pooled and the results (from the mean of the ten datasets) were combined to obtain estimates of parameters and standard errors. These estimates then correctly reflected both sampling variability and the additional uncertainty due to missing data and imputation.

Missing data for the other mediators—improvement of understanding (1.6 %) and improvement in self-confidence (1.0 %)—were not imputed, but because they are endogenous variables (i.e., they are caused by one or more variables in the model), these cases were still included in the analyses. All missing values at endogenous variables were assumed to be missing at random.

Individuals with missing data in the independent (exogenous) variables—affective quality (1.9 %), information provision (7.6 %), and shared decision-making (4.8 %)—were not included in the analyses, because missing data on these variables indicate no experiences with this particular communication aspect (code 5 on the experience scale: no experience/don’t know).

#### Steps in analyses

First, we described the characteristics and study outcomes of all participating adolescents. Second, we estimated the association between independent variables (affective quality, information provision, and shared decision-making) and the dependent variable (TDS after 1 year) by means of linear regression, separately for each independent variable. To assess 1-year changes in psychosocial problems, we adjusted for TDS at baseline. We further adjusted for the following potentially confounding variables: age, gender, parental employment, and care setting. Third, we explored the direct and indirect, i.e., mediated, associations between attributed relevance—actual experience discrepancies regarding communication and 1-year changes in psychosocial problems using structural equation modelling (SEM) with maximum likelihood estimation [29]. The dependent variable (TDS after 1 year) in the SEM model was always adjusted for TDS at baseline and the afore-mentioned confounding variables. We assessed the potential mediating roles of treatment adherence, improvement of understanding, and improvement in self-confidence in the associations between relevance-experience discrepancies and changes in psychosocial problems after 1 year. Residual correlations between pairs of all three mediators were included in the model, to account for non-independencies among the mediator variables.

Three different SEM analyses were performed separately for each independent variable (affective quality, information provision, and shared decision-making). Descriptive analyses were performed using SPSS version 20 and the structural equation analyses in Mplus version 7.1.

## Results

### Participant characteristics

Adolescent and family characteristics and care-related characteristics of the study sample are presented in Table 1. Most of the 315 patients in this sample received psychosocial care from a mental health care organization (76.8 %).

**Table 1:** Participant characteristics (*n* = 315)

Adolescent and family characteristics	
Age; mean (standard deviation)	15.2 (1.7)
Gender (female); %	61.3
Ethnicity (Dutch); %	89.1
Family composition (two-parent family); %	46.0
Parental employment (at least one parent employed); %	77.8
Care-related characteristics	
Care setting; %	
Child and adolescent social care	23.2
Child and adolescent mental health care	76.8
Care and treatment trajectory; %	
Start	
Within 3 months	88.3
After 3 months	11.7
End	
Within 3 months	43.8
Within 3–12 months	24.8
Not completed after 12 months	31.4
Duration	
Less than 6 months	45.8
6 months or more	54.2

For some patients treatment did not start within the first 3 months after registration (11.7 %). This was due to waiting lists and to an initial focus of care and communication on the parents or other family members. About one third of the sample patients were still in contact with the professional 1 year after entry (31.4 %). Over half of the sample received care and treatment for 6 months or more (54.2 %).

### Patient-professional communication and outcomes

Table 2 presents separately the study’s outcomes for all communication aspects and categories. In general, results show less reduction of psychosocial problems and poorer mediation outcomes when a communication aspect was considered to be important but was subsequently less experienced (unmet communication needs).

**Table 2:** Scores on outcomes and mediators for the three independent variables: frequencies (*n*, %), means (M) and standard deviations (SD)

Independent variables (attributed relevance versus actual experience)	*n* (%)^a^	Dependent variables			Mediating variables		
		TDS at baseline (T1)	TDS after 1 year (T3)	TDS change scores (T3–T1)	Treatment adherence	Improvement of understanding	Improvement in self-confidence
		*M* (SD)	*M* (SD)	*M* (SD)	*M* (SD)	*M* (SD)	*M* (SD)
Affective communication quality							
Agreement	140 (61.4)	14.4 (4.8)	11.5 (4.7)	−2.9 (4.3)	7.5 (1.9)	6.5 (2.0)	6.1 (2.1)
Important—less experienced	42 (18.4)	15.6 (5.3)	13.9 (5.3)	−1.7 (5.6)	6.5 (2.3)	5.2 (2.1)	5.0 (2.3)
Less important—experienced	46 (20.2)	15.7 (5.1)	12.8 (4.9)	−2.9 (3.7)	7.4 (1.9)	6.8 (1.8)	6.0 (2.3)
Information provision							
Agreement	145 (68.4)	14.7 (5.3)	11.6 (5.0)	−3.1 (4.4)	7.4 (1.9)	6.6 (1.8)	6.2 (2.0)
Important—less experienced	27 (12.7)	15.4 (4.7)	14.9 (4.5)	−0.5 (3.9)	7.3 (2.2)	5.6 (2.0)	5.5 (2.1)
Less important—experienced	40 (18.9)	16.2 (3.6)	13.1 (4.6)	−3.0 (4.5)	7.2 (2.0)	6.3 (2.2)	5.9 (2.1)
Shared decision-making							
Agreement	157 (71.0)	15.1 (5.1)	12.2 (4.8)	−2.9 (4.4)	7.6 (1.8)	6.7 (1.7)	6.1 (2.1)
Important—less experienced	33 (14.9)	15.3 (5.2)	13.9 (5.5)	−1.4 (4.6)	6.3 (2.5)	4.7 (2.5)	4.9 (2.6)
Less important—experienced	31 (14.0)	14.4 (4.5)	11.8 (5.1)	−2.6 (4.4)	6.7 (2.2)	6.3 (2.1)	5.9 (1.9)
Total	252 (100.0)	15.0 (5.0)	12.3 (5.0)	−2.7 (4.4)	7.2 (2.1)	6.2 (2.1)	5.8 (2.2)

*TDS* total difficulties score

^a^Numbers do not add up to *n* = 315 due to missing values. Cases were only included if values for TDS at baseline and TDS after 1 year were available

Average adolescents’ mean TDSs were lower at 1 year follow-up than at baseline, indicating positive changes. However, the smallest reduction was found for adolescents with unmet communication needs. These patients had a reduction of 0.5–1.7 points on the TDS after 1 year compared to reductions of 2.6–3.1 points for patients whose attributed relevance and actual experience regarding communication were in agreement or who experienced communication that they rated less important.

Regarding possible mediators, in general the poorest scores were also found for adolescents with unmet needs. The only exception was adolescents who had unmet needs regarding information provision but who had similar treatment adherence as the other two groups.

### Direct and indirect associations between patient-centered communication and reduction of psychosocial problems after 1 year

**Fig. 1:**
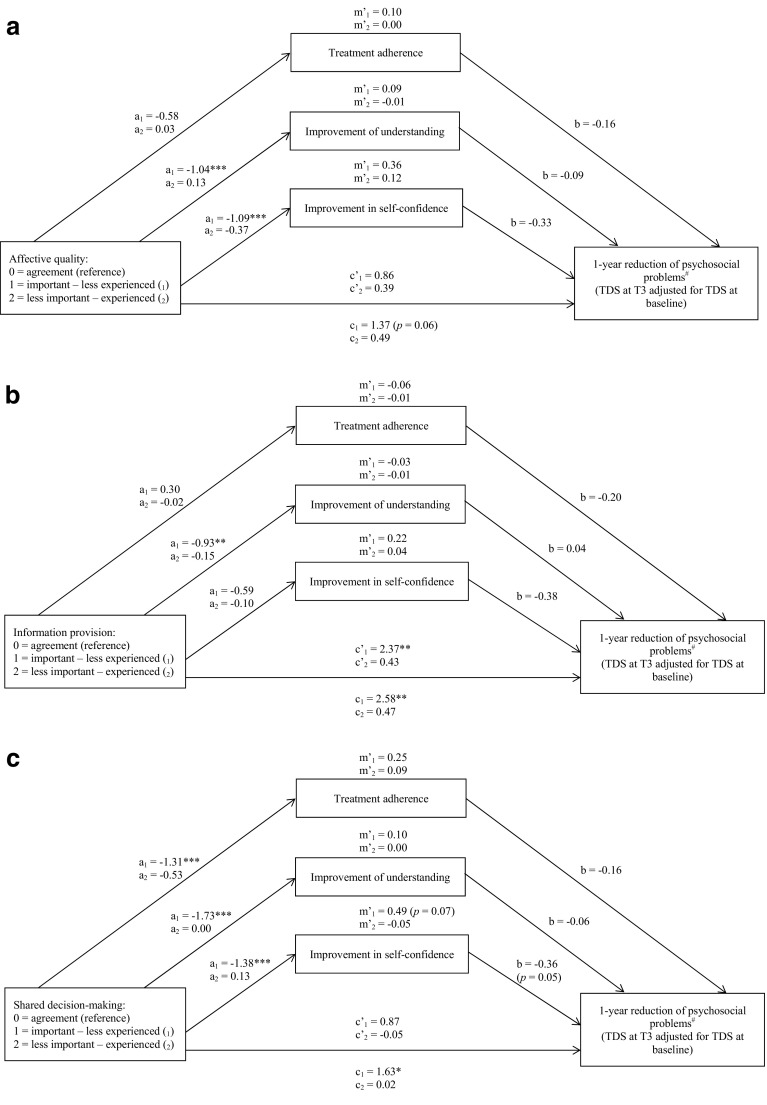
**a** Results of mediation analyses of affective quality of the communication: direct and indirect effects. **b** Results of mediation analyses of information provision: direct and indirect effects. **c** Results of mediation analyses of shared decision-making: direct and indirect effects. *p < 0.05; **p < 0.01; ***p < 0.001. a1 Direct associations between important—less experienced communication and mediators. , a2 Direct associations between less important—experienced communication and mediators. b Direct associations between mediators and reduction of psychosocial problems after 1 year. c1 Direct associations between important—less experienced communication and reduction of psychosocial problems after 1 year, adjusted for age, gender, parental employment, care setting. c2 Direct associations between less important—experienced communication and reduction of psychosocial problems after 1 year, adjusted for age, gender, parental employment, care setting. c′1 Direct associations between important—less experienced communication and reduction of psychosocial problems after 1 year, adjusted for age, gender, parental employment, care setting, and mediators (treatment adherence, improvement of understanding, and improvement in self-confidence). c′2 Direct associations between less important—experienced communication and reduction of psychosocial problems after 1 year, adjusted for age, gender, parental employment, care setting, and mediators (treatment adherence, improvement of understanding, and improvement in self-confidence). m′1 Indirect associations of important—less experienced communication and reduction of psychosocial problems after 1 year via each mediator separately, adjusted for age, gender, parental employment, care setting. m′2 Indirect associations of less important—experienced communication and reduction of psychosocial problems after 1 year via each mediator separately, adjusted for age, gender, parental employment, care setting. m′1 and m′2 do not always correspond to the multiplication of a1i × bi and a2i × bi as estimates are averages of a series of analyses due to the multiple imputation procedure. ^#^ Higher scores indicate more psychosocial problems, thus less improvement

Figure 1a–c show the direct and indirect, i.e., mediated, associations between attributed relevance—actual experience discrepancies regarding communication, and reduction of psychosocial problems after 1 year. Regarding *affective quality of the communication* the analysis revealed a marginally significant direct association between unmet needs for affective quality (i.e., the affective quality of the communication was considered to be important but then not experienced) and less reduction of psychosocial problems after 1 year (*p* = 0.06). This association weakened after adjusting for possible mediating variables; no statistically significant mediating effects were found. Regarding *information provision* the analysis revealed a significant direct association between unmet needs and less reduction of psychosocial problems. This association remained after adjusting for the possible mediating variables, and no statistically significant mediating effects were found. Regarding *shared decision*-*making* the analysis revealed that the association between unmet needs and less reduction of psychosocial problems was partly mediated by less improvement in self-confidence. In other words, adolescents with unmet communication needs had 1.63 points less reduction on their TDS than adolescents whose needs and experiences regarding communication matched. Approximately 30 % of these 1.63 points (estimate = 0.49 with a *p* value of 0.07) can be explained by the negative effect of patients’ unmet communication needs on the improvement in their self-confidence, which in turn leads to less reduction of their psychosocial problems.

## Discussion

This study demonstrates that adolescents’ unmet communication needs—they considered communication to be important but it was not experienced—have a negative impact on the reduction of psychosocial problems 1 year after the start of psychosocial care. No effects were found for adolescents who did not express a need for specific communication domains but nevertheless experienced it. Regarding shared decision-making, the association between unmet needs and less reduction of psychosocial problems was partly mediated by less improvement in self-confidence. There were no mediation effects for associations between affective quality and information provision with a reduction of psychosocial problems after 1 year.

Adolescents’ unmet communication needs negatively affected the reduction of their psychosocial problems after 1 year. This finding aligns with research conducted in other health care settings, which showed positive effects of patient-centered approaches on care outcomes. For example, in preventive child health care a family centered approach has been shown to contribute to more and earlier identification of risks for social-emotional problems, and to the identification of families that need additional care [30]. Although patient-centered communication may have a positive effect on health outcomes, evidence as to these associations is very limited [31, 32]. One reason may be that most studies adopt a ‘one way fits all’ approach that fails to take individual patients’ needs into account, whereas we know that not all patients want the same thing [33]. This study used a tailoring approach in measuring patient-centered communication by combining patients’ attributed relevance to communication at the start of treatment and their actual communication experiences during treatment; this may explain our findings.

Adolescents who did not express a need for specific communication domains, but nevertheless experienced it, had an improvement in psychosocial problems similar to that of adolescents whose experiences matched their attributed relevance. Although we previously found an association between this type of discrepancy regarding affective quality of the communication and less improvement in self-confidence [18], in the present study that association had no statistical significance. The reason is that in the present study ‘improvement in self-confidence’ was taken into account as a continuous variable, whereas in the previous study we were interested in a specific group, and therefore, dichotomized the variables. This may indicate that such an effect holds only for adolescents with a particularly low level of improvement in self-confidence, and is not gradual. We are not aware of any other literature specifically describing how this type of discrepancy between attributed relevance to communication and communication experiences affects outcomes of care.

Unmet needs for shared decision-making were significantly related to less reduction of psychosocial problems, but partly mediated by improvement in self-confidence. Studies performed in other health care settings reported positive effects of shared decision-making on patients’ self-efficacy skills, empowerment, and confidence [34–36], also in mental health care settings [37, 38], though the latter is still limited. Involving patients in decisions regarding their own care may increase their feelings of being an equal partner, and their trust in the decisions made. In turn, this may strengthen their self-esteem and confidence, and empower them to handle their problems and their own care process [39, 40]. This may in turn lead to better health outcomes. However, we should keep in mind that this was found when patients display a high need for shared decision-making and that not all patients want to be involved in the decision-making process [33, 41].

Unmet needs for affective quality were only marginally related to less reduction of psychosocial problems. This was unexpected because affective quality of the communication is considered by adolescents in psychosocial care to be the most important communication domain [33], and these needs are often not met [18]. A reason for our finding may be that affective quality has its highest impact on outcomes in the first stage of the care process. Furthermore, there may be other mechanisms at work, such as motivation, social support, or trust in the system [42].

Unmet needs for information provision were significantly related to less reduction of psychosocial problems; this association was hardly mediated by treatment adherence, improvement of understanding, and improvement in self-confidence. This finding aligns with research among surgery patients; associations of matching needs and experiences regarding information provision were linked with less depression, less anxiety, better coping, and better patient satisfaction [41]. We are not aware of literature describing potential mediators in these associations. We previously found an association between unmet needs for information provision and less improvement of understanding [18], but in our study this was no mediator for the association between unmet needs and less reduction of psychosocial problems. This indicates an independent effect of unmet needs for information provision on improvement of understanding and reduction of psychosocial problems. As with what we found regarding affective quality of the communication, in these associations there may be other mechanisms working that were not taken into account in our study.

This study has considerable strengths. First, we studied patient-centered communication by comparing patients’ attributed relevance regarding communication before the start of the care process with their actual communication experiences. Second, we distinguished three communication functions and analyzed their relationships with psychosocial problems; to identify pathways between communication and outcomes we used a longitudinal study with multiple assessments to study the possible mediating effects of treatment adherence, improvement of understanding, and improvement in self-confidence. Third, in our analysis we took into account the correlations between the three mediators instead of treating them as independently measured variables.

This study also has some limitations. First, the answers of professionals concerning their patients’ improvements may be subject to social desirability because these have to do with the effects of their own treatment. However, by also including the patient’s perspective we reduced possible information bias. Second, we could not address the direction of pathways between the mediators as we measured them simultaneously. This requires additional study.

Repeated measurements may in future studies help us to gain more evidence as to the causal relations between patient-centered communication and different outcomes during the care process. For example, it would be better to measure patients’ communication needs repeatedly during the care process because these needs may change over time during and due to treatment. Also, future studies may include other possible mediators such as motivation, social support, or trust in the system [42, 43]. More insight into these pathways will contribute to the improvement of professionals’ communication skills.

This study provides empirical evidence for the importance of patient-centered communication in adolescent psychosocial care. Professionals should be aware of the negative effects on care outcomes when patients’ communication needs are not met. They need to take very seriously the negative impact on patients’ self-confidence when their needs for shared decision-making are not met, as this seems to lead to less reduction of their psychosocial problems. Our findings provide major opportunities to increase the effectiveness of psychosocial care for children and adolescents.

## Electronic supplementary material

Below is the link to the electronic supplementary material.

Supplementary material 1 (DOCX 16 kb)
