# Supplementary material for: Less reduction of psychosocial problems among adolescents with unmet communication needs
Source: Eur Child Adolesc Psychiatry. 2016 Sep 13;26(4):403–12. doi: 10.1007/s00787-016-0901-y (PMC5364263; doi:10.1007/s00787-016-0901-y)
Supplement: Supplementary file 1 — Supplementary material 1 (DOCX 16 kb) [file 787_2016_901_MOESM1_ESM.docx]

**Supplement 1. Original Dutch questionnaire concerning adolescents’ communication needs^a^  with English translations^b^**

| **Affective quality of the communication** | | | |
| --- | --- | --- | --- |
| *Naar mijn mening moeten hulpverleners…* | | *In my opinion care professionals should…* | |
| … | mij altijd serieus nemen. | … | always take me seriously. |
| … | altijd genoeg tijd voor mij nemen. | … | always take enough time for me. |
| … | zich aan beloftes houden. | … | keep their promises. |
| … | mij respecteren. | … | respect me. |
| … | vertrouwelijk omgaan met mijn gegevens. | … | treat information about me confidentially. |
| … | begrijpen wat mijn problemen of klachten zijn. | … | understand what my problems or complaints are. |
| … | aandachtig naar mij luisteren. | … | listen to me attentively. |
| … | mij op mijn gemak stellen. | … | make me feel comfortable. |
| … | mij aanmoedigen over mijn problemen te praten. | … | encourage me to talk about my problems. |
| **Information provision** | | | |
| *Naar mijn mening moeten hulpverleners…* | | *In my opinion care professionals should…* | |
| … | voldoende voorlichting geven over wat ik aan de hulpverlening kan hebben. | … | provide sufficient information on how I can benefit from care. |
| … | uitgebreide uitleg geven over de aanpak bij deze hulpverlening. | … | give detailed explanation of the approach to this care. |
| … | mij informeren hoe ik met de problemen kan omgaan. | … | inform me on how I can deal with my problems. |
| … | mij informatie geven over verschillende hulpverleningsmogelijkheden. | … | provide information about different possibilities for care. |
| … | mij informatie geven over andere vormen van hulpverlening of ondersteuning zoals bijvoorbeeld zelfhulpprogramma’s, jongerenondersteuning, patiëntenverenigingen, E-hulp of alternatieve geneeswijzen. | … | Provide information about other types of care or support such as self-help programs, youth support, patient organizations, E-care or alternative medicine. |
| **Shared decision-making** | | | |
| *Naar mijn mening moeten hulpverleners…* | | *In my opinion care professionals should…* | |
| … | rekening houden met mijn voorkeuren voor een bepaalde vorm van hulpverlening. | … | consider my preferences for a specific type of care. |
| … | bereid zijn met mij te praten over zaken die naar mijn mening niet goed zijn verlopen. | … | be willing to talk to me about things that I believe have not been successful. |
| … | mij de mogelijkheid geven om bepaalde dingen zelfstandig te regelen. | … | give me the possibility to manage certain things independently. |
| … | geen advies of behandeling aan me opdringen. | … | not impose advice or treatment on me. |
| … | samen met mij doelen opstellen en samen met mij een stappenplan maken voor de hulpverlening. | … | set goals and plans for the care process in collaboration with me. |

^a^ Adolescents rated the same items to assess their actual experiences, for example: “The care professional considered my preferences for a specific type of care.”, “The care professional always took me seriously.”, “The care professional provided sufficient information on how I can benefit from care.”

^b^ This questionnaire is an adapted version of the Consumer Quality Index (CQI) [20]. Questions were derived from three existing CQI versions that have been used in preventive child health care [21], outpatient mental health care [22, 23], and outpatient occupational therapy [24].
